# Pediatric Chronic Intestinal Failure: Something Moving?

**DOI:** 10.3390/nu16172966

**Published:** 2024-09-03

**Authors:** Aysenur Demirok, Sjoerd C. J. Nagelkerke, Marc A. Benninga, Cora F. Jonkers-Schuitema, Suzanne M. C. van Zundert, Xavier W. Werner, Bruno Sovran, Merit M. Tabbers

**Affiliations:** 1 Pediatric Gastroenterology and Nutrition, Emma Children’s Hospital, Amsterdam University Medical Center, University of Amsterdam, 1105 AZ Amsterdam, The Netherlandss.m.vanzundert@amsterdamumc.nl (S.M.C.v.Z.);; 2 Tytgat Institute for Liver and Intestinal Research, Amsterdam Gastroenterology Endocrinology Metabolism, Academic Medical Center, University of Amsterdam, 1105 BK Amsterdam, The Netherlands; 3 Department of Pediatric Surgery, Emma Children’s Hospital, Amsterdam University Medical Center, University of Amsterdam, Meibergdreef 9, 1105 AZ Amsterdam, The Netherlands; 4 Emma Center for Personalized Medicine, Amsterdam University Medical Center (UMC), University of Amsterdam, 1105 AZ Amsterdam, The Netherlands; 5 Amsterdam Reproduction and Development and Amsterdam Gastroenterology Endocrinology Metabolism Research Institutes, 1105 AZ Amsterdam, The Netherlands

**Keywords:** catheter-related bloodstream infection, catheter-related thrombosis, central venous catheter, congenital enteropathy, home parenteral nutrition, intestinal failure, microvillus inclusion disease, pediatric, pediatric intestinal pseudo-obstruction, short bowel syndrome

## Abstract

Pediatric chronic intestinal failure (PIF) is a rare and heterogeneous condition characterized by the inability of the patient’s intestine to adequately absorb the required fluids and/or nutrients for growth and homeostasis. As a result, patients will become dependent on home parenteral nutrition (HPN). A MEDLINE search was performed in May 2024 with keywords “intestinal failure”, “parenteral nutrition” and “pediatric”. Different underlying conditions which may result in PIF include short bowel syndrome, intestinal neuromuscular motility disorders and congenital enteropathies. Most common complications associated with HPN are catheter-related bloodstream infections, catheter-related thrombosis, intestinal failure-associated liver disease, small intestinal bacterial overgrowth, metabolic bone disease and renal impairment. Treatment for children with PIF has markedly improved with a great reduction in morbidity and mortality. Centralization of care in specialist centers and international collaboration between centers is paramount to further improve care for this vulnerable patient group. A recently promising medical therapy has become available for children with short bowel syndrome which includes glucagon-like peptide 2, a naturally occurring hormone which is known to delay gastric emptying and induce epithelial proliferation. Despite advances in curative and supportive treatment, further research is necessary to improve nutritional, pharmacological and surgical care and prevention of complications associated with parenteral nutrition use.

## 1. Introduction: Definitions, Prevalence and Goal of Treatment

Intestinal failure is commonly defined as “the reduction of gut function below the minimum necessary for the absorption of macronutrients and/or fluids and electrolytes, such that intravenous supplementation is required to maintain health and/or growth” [1,2]. According to the duration of the disorder, intestinal failure is classified as type I; acute intestinal failure requiring intravenous support for days to weeks, type II; prolonged acute intestinal failure requiring intravenous support for weeks to months and type III; and pediatric chronic intestinal failure (PIF) where patients are in a stable clinical condition and are dependent on intravenous support for months to years [1]. Recent studies show a prevalence of home parenteral nutrition (HPN) use in children ranging from 9.6 to 27 children per million [3,4,5].

In developed countries, the most prevalent underlying conditions leading to PIF in children are (1) short bowel syndrome, (2) intestinal neuromuscular motility disorders and (3) congenital enteropathies [2,6]. Children suffering from PIF who are clinically stable and are expected to be dependent on parenteral nutrition for at least three months preferably receive their parenteral nutrition at home. Parents/caregivers should be able to cope with all technical, medical and emotional aspects of HPN and will be trained simultaneously in the hospital before discharge. If needed, home nursing and/or social help should also be arranged prior to discharge [7]. Since patients receiving HPN are at risk of several life threatening complications, the goal of treatment in PIF is to wean patients from parenteral nutrition and achieve enteral autonomy whilst ensuring growth and homeostasis, and preventing the development of complications [8,9]. Weaning from parenteral nutrition is achieved in approximately 50% to 80% of all pediatric patients with PIF, with the majority of patients having short bowel syndrome as opposed to other causes of intestinal failure [2,10,11,12,13].

## 2. Underlying Conditions

### 2.1. Short Bowel Syndrome

Short bowel syndrome is the most prevalent underlying cause of PIF, accounting for 50% to up to 80% of patients receiving HPN [2,10,11,14]. In children, short bowel syndrome is caused by congenital or acquired loss of a major part of the small intestine, most often due to necrotizing enterocolitis, mid-gut volvulus, gastroschisis, or intestinal atresia [15,16]. The incidence of short bowel syndrome is reported to be present in 24.5/100,000 live births, with a higher incidence in preterm born children compared to term born infants [17]. The key aspects of management focus on maintaining fluid and nutrient balance using enteral and parenteral nutrition to ensure adequate growth and promoting intestinal adaptation [18]. Parenteral nutrition is usually started in the immediate postoperative period and additional enteral feeding is advised to be introduced early, if tolerated, to reduce the duration of parenteral nutrition [19]. A higher percentage of small intestinal length and presence of the ileocecal valve have been identified as positive predictive characteristics for weaning from parenteral nutrition in retrospective studies [20,21,22,23,24,25]. Weaning rates from parenteral nutrition for children suffering from short bowel syndrome are reported to be between 60% and 80% [10,11,12,13,20,21,22]. Most children wean from parenteral nutrition within one year after intestinal resection; however, weaning after four years or later is described as well. If weaning from parenteral nutrition is unsuccessful, surgical therapies can be considered including bowel lengthening procedures. The two most performed surgical bowel lengthening procedures are the longitudinal intestinal lengthening and tailoring (LILT) procedure and serial transverse enteroplasty (STEP) procedure, which were first described in 1980 and 2003, respectively [26,27]. These procedures are performed on dilated bowel loops of children and result in reduced bowel dilation and increased bowel length. A recently performed systematic review describing the success rates of these procedures reported fair to poor results for both procedures [28].

### 2.2. Intestinal Neuromuscular Motility Disorders

Intestinal neuromuscular motility disorders are on the most severe end of the spectrum of gut motility disorders and comprise a group of disorders with the best known being pediatric intestinal pseudo-obstruction (PIPO), total colonic aganglionosis and Hirschprung disease [29]. PIPO is characterized by the chronic (>2 months from birth or > 6 months thereafter) inability of the gastrointestinal tract to propel its contents, mimicking mechanical obstruction in the absence of any lesion occluding the gastrointestinal tract [30]. This disease presents with great heterogeneity during the clinical course within and between patients [30]. Presenting symptoms include abdominal distension, bilious vomiting and failure to pass stools. Involvement of the urinary tract with signs as megacystis is described in 20% of patients [31]. An incidence of approximately 1 in 40,000 live births has been reported by a study in the United States [32]. A Japanese study revealed a point prevalence of 3.7 in one million children under the age of 15 years Genetic testing has identified a multitude of pathogenic mutations with mutations in the gene encoding for the enteric smooth muscle actin–gamma 2 (ACTG2) being the most known [33]. Scant evidence is available with regard to pharmacological therapies for PIPO where adult data and case reports make up the majority of available data. The serotonin 5HT_4_ receptor agonist Cisapride has shown to improve small intestinal motility and feeding tolerance but has been withdrawn from the market in many countries due to serious cardiac side effects [30,34,35]. Other prokinetic drugs such as erythromycin, metoclopramide, domperidone, octreotide and neostigmine have mostly been studied in adult populations [30,36,37,38]. Octreotide has been shown to increase enteral feeding through promoting the intestinal absorption with ≥ 10 mL/kg/day in 7 out of 16 (44%) patients in one study of children with PIPO of whom three tolerated full enteral feeds and weaned completely off TPN [36]. Although the mechanism by which octreotide increases the tolerance of enteral feeds is not well understood, an increase in intestinal motility is found to be induced by octreotide [36].

Nutritional management plays a pivotal role in PIPO. Various strategies to maintain nutrition in patients with PIPO include oral, enteral and parenteral nutrition. Due to its heterogenous clinical entity, each patient requires a tailor-made nutritional management. Dependency on partial or total HPN ranges between 24% and 70% during the disease course in several PIPO cohorts, with a duration from 3 months up to life long [31,33,39,40,41,42].

Surgery has an important role in PIPO but it should be emphasized that only necessary procedures should be undertaken to reduce the risk of adhesion formation and prolonged ileus after surgery [43]. The formation of a gastrostomy, jejunostomy or ileostomy provides gastrointestinal decompression resulting in a reduction in the frequency of vomiting, abdominal distension and an increase in health-related quality of life [39,40,43]. However, ostomy-associated complications (e.g., prolapse) remain significant, thus it should be carefully constructed and managed [41]. The rarity and complexity of this disease leads to serious diversity in management strategies even among European intestinal failure teams [42]. Recently reported mortality rates vary from 4.8% to 18.2%, mostly due to sepsis, multi-organ failure or malnutrition [31,44]. Due to the diagnostic and therapeutic challenges of this rare disease, centralization of care including treatment in a multidisciplinary expertise team is strongly recommended.

### 2.3. Congenital Enteropathies

Congenital enteropathies are disorders leading to severe intestinal failure and harmful electrolyte disbalances and dehydration due to severe watery diarrhea. These disorders include microvillus inclusion disease (MVID), tufting enteropathy (TE) and congenital diarrheal disorders (for example neurogenin-3 enteric endocrinopathy, congenital sodium diarrhea, congenital chloride diarrhea) [2,45,46]. Currently, no cure exists for these disorders which seem to inherit in an autosomal recessive manner [6,47,48] In MVID, biopsies of the intestine show atrophy of the microvillus brush border concomitant with microvillus inclusions in the enterocytes cytoplasm [49]. Mutations in the MYO5B gene have been found to be causative for MVID in a subset of patients [50,51]. Histopathology in TE shows villous atrophy, crypt hyperplasia and densely packed enterocytes in epithelial tufts. Mutations in the genes encoding for EPCAM and SPINT2 have been reported as causative for TE [47,52,53]. Epidemiological data on incidence and prevalence of these disorders are lacking. In studies with large cohorts of PIF, it is described that around 10% of children on HPN are suffering from a congenital enteropathy [10,11]. Since no cure exists for these patients, the mainstay of treatment is supportive, lifelong therapy with HPN. Laboratory research is performed in various models in order to find a cure for these disorders [47,48].

All parents of a child suffering from intestinal neuromuscular disorders and congenital enteropathies should be offered consultation with a geneticist in order to provide counseling with regards to future pregnancies.

## 3. Current Treatment of Pediatric Intestinal Failure

### 3.1. Multidisciplinary Care

In order to reduce the risk of complications and mortality, it has been well established that care for children with PIF should be provided by specialist centers with a multidisciplinary team focused on intestinal rehabilitation and HPN [2,54]. Therefore, early referral to an expert center, which houses an intestinal rehabilitation program and collaborates with or has an intestinal transplantation program is advocated. International collaboration between centers should actively be sought to improve both care and research. The multidisciplinary team should offer 24 h phone support and should include at least a pediatric gastroenterologist, pediatric surgeon, pediatric nurse with experience in central venous catheter management, a pediatric dietician, a child psychologist and a pharmacist [7,54,55] After discharge, children should be seen at least four times a year in the outpatient clinic, with infants often being required to visit more frequently. The updated guidelines on pediatric HPN by the European Society of Paediatric Gastroenterology, Hepatology and Nutrition (ESPGHAN) and the European Society for Clinical Nutrition and Metabolism (ESPEN), supported by the European Society of Paediatric Research (ESPR) together with the Chinese Society of Parenteral and Enteral Nutrition (CSPEN) can be utilized for planning of clinical and laboratory assessment [7].

### 3.2. Home Parenteral Nutrition

Parenteral nutrition at home is the mainstay of therapy for all children suffering from PIF, regardless of their underlying disorder. In recent years, the incidence of HPN use in children increased due to improvements in neonatal, nutritional, surgical, and catheter care [7,56]. The goal of treatment in PIF is to wean patients from HPN and achieve enteral autonomy whilst ensuring growth and homeostasis and preventing the development of complications.

The guidelines on pediatric parenteral nutrition state that parenteral nutrition admixtures, also called individualized bags, should be personalized and tailored for children with PIF to suit a specific patient. Standardized bags include standardized formulations compounded by pharmaceutical companies. Use of standardized bags should be avoided if patients receive long-term HPN [57]. However, a recent study, found that children with PIF over 2.5 years of age receiving standardized parenteral nutrition bags showed a comparable gain in weight and height when compared to children receiving individualized bags. Furthermore, children on standardized parenteral nutrition bags did not show an increase in electrolyte disturbances or biochemical abnormalities over a two-year period [58]. The usage of standardized parenteral nutrition bags has several possible advantages, including a longer shelf life and cost reduction [58,59]. Especially licensed industrially prepared ready-to-use multi chamber bags, which are standardized PN bags with compartments containing nutrients, may reduce the complexity of prescribing and preparing individualized PN [60]. It can be advised to use standardized parenteral nutrition bags if this is possible considering the need.

### 3.3. Medical Management

Various medical therapies exist for PIF. Table 1 gives an overview of current medications, their indications for use, level of evidence and references. The classes of medications include: antisecretory, adjunctive absorptive, prokinetic, antibiotic and probiotic agents and growth factors.

### 3.4. Intestinal Transplantation

In Europe, intestinal transplantation is seen as a last resort of treatment of children suffering from PIF with indications of loss of venous access, recurrent episodes of life threatening sepsis (for example fungal) and IFALD [82]. The rate of intestinal transplantation has declined since 2008, mostly due to improvements in nutritional, surgical and medical care of patients on HPN [83,84]. Five year survival for children receiving an isolated intestinal or multivisceral transplant in experienced high-volume centers is around 70%, which is inferior to survival rates in children receiving long-term HPN with survival rates exceeding 90% [7,10,11,83,85,86,87]

## 4. Complications of Long-Term Home Parenteral Nutrition

Children with PIF receiving HPN are at risk of several complications which will be discussed separately.

### 4.1. Central Venous Catheter-Related Bloodstream Infection

Catheter-related bloodstream infections (CRBSIs) are an important and potentially lethal complication of central venous catheter usage. Parents are instructed to contact the hospital if their child develops a temperature >38.5 °C or has other symptoms suggestive of infection. Cultures taken both peripherally and from the central venous catheter should be drawn prior to administration of broad acting antibiotics covering both Gram-positive and Gram-negative pathogens [7,88]. As the central venous catheter of children suffering from PIF is regarded as a lifeline, efforts should be undertaken to treat CRBSI prior to removal of the catheter. Repeated catheter removal is not desirable due to the risk of loss of suitable vessels. However, removal might be inevitable in the presence of clinical deterioration or additional complications (such as thrombophlebitis, endocarditis and bloodstream infection that continues despite > 72 h of antimicrobial therapy to which the agents are susceptible), persisting or relapsing bacteremia or for particular agents such as Staphylococcus aureus, Pseudomonas species, Candida or mycobacteria [89,90]. However, a recent retrospective study in children on HPN diagnosed with a Staphylococcus aureus bloodstream infection found a successful catheter salvage rate of 100%, suggesting that catheter salvage can be attempted after careful consideration by an expertise team [91]. Several antimicrobial line locks such as taurolidine are used to prevent CRBSI in children with PIF. Taurolidine locks have shown to greatly reduce the incidence of CRBSI. A retrospective study reported 8.6 CRBSI per 1000 catheter days in children receiving a heparin lock compared to 1.1 CRBSI per 1000 catheter days in the taurolidine group. No adverse effects were reported [92]. In another retrospective study in 37 neonates, taurolidine was successfully used both for prevention and treatment of catheter-related bloodstream infections, without major adverse effects [93]. Data from this study and several adult studies have led to the recommendation to use taurolidine locks during long-term central venous catheter usage [88,94]. Microbial adaptation to taurolidine has not yet been reported in patients using long-term taurolidine locks [95].

### 4.2. Catheter-Related Thrombosis

The current ESPGHAN guideline on pediatric parenteral nutrition states that there is insufficient evidence to advocate the prophylactic use of anticoagulants in children receiving HPN to reduce catheter-related thrombosis, occlusion and infection [88]. This leads to great heterogeneity in strategies concerning thrombosis, as 46% of the intestinal failure teams in Europe use prophylactic anticoagulation while the others do not use it standardly [96].

A study revealed an incidence of central venous thrombosis of 57% in a cohort of children on home PN where 40% of patients had two or more thrombosed central veins [11]. Recurrent thrombosis poses a significant barrier for PN infusion due to higher risk of loss of vascular access [12].

In children on HPN, three studies have been performed to investigate the efficacy and safety of prophylactic anticoagulants to prevent catheter thrombosis. In 2003, Newall et al. studied the effect of warfarin therapy and showed that mean catheter duration increased from 160.9 days to 352.7 days in eight children with short bowel syndrome. Bleeding complications did not occur [97]. Vegting et al. studied the effect of low molecular weight heparin (LMWH) and vitamin K antagonist prophylaxis [98]. Per 1000 PN days, the non-prophylaxis and prophylaxis groups had 2.6 and 0.1 occlusions (*p* = 0.04) and 4.6 and 2.1 infections (*p* = 0.06), respectively. Bleeding complications did not occur [98].

Another study of Nagelkerke et al. found an incidence of catheter-related thrombosis on prophylactic anticoagulation of 0.2 per 1000 catheter days, without bleeding complications [99]. Prospective, international studies including both patients on and off prophylactic anticoagulation should be performed to assess efficacy and safety of prophylaxis.

### 4.3. Intestinal Failure-Associated Liver Disease (IFALD)

IFALD is a serious complication occurring in around 20% to 33% of patients receiving HPN [10,100,101,102]. It is defined as “hepatobiliary dysfunction as a consequence of medical and surgical management strategies for PIF, which can variably progress to end-stage liver disease, or can be stabilized or reversed with promotion of intestinal adaptation” [103]. Both nutrition related factors (e.g., lack of oral/enteral nutrition and the type and amount of lipid emulsion infused) as well as patient-related factors such as prematurity, recurrent episodes of sepsis and CRBSI are reported as risk factors leading to IFALD [103,104,105]. Currently, it is unclear how to best diagnose and stage IFALD in clinical practice as its reference standard is histology which is not practical to use in routine care. Studies investigating biochemical and non-invasive measurement techniques such as transient elastography to diagnose and stage IFALD have been undertaken but so far have not been able to identify a usable technique and cut-off value [103,106,107,108]

Lipid emulsions are regarded as the main parenteral nutrition related factor leading to liver injury in patients suffering from PIF. Contributing factors, which are known to induce liver injury, include high levels of Ω-6 fatty acids and phytosterols and relatively low α-tocopherol levels, which is a strong antioxidant. Soybean oil lipid emulsions, containing high concentrations of Ω-6 fatty acids such as linoleic acid and relatively low concentrations of α-tocopherol, seems to be correlated with development of cholestatic liver disease [103,105]. Nutritional therapies, where lipid emulsions containing pure or partly fish oil (with high Ω-3 fatty acids and the antioxidant α-tocopherol and reduced Ω-6 fatty acids and phytosterol) are used, show successful resolution of cholestasis in patients with PIF and liver disease [109,110]. Although high quality evidence is lacking, composite fish-oil lipid emulsions seem to have some advantages over conventional lipids in neonates and pediatric patients due to their hepatoprotective characteristic [111,112,113,114,115,116]. However, long-term administration of pure fish oil only is not recommended as it might result in linoleic acid insufficiency [117]. Temporary reduction or cessation of lipid infusion should be considered in case of persistent cholestasis and essential fatty acids should be monitored in these cases. It is of great importance to prevent essential fatty acid deficiency as these are crucial for brain development, especially in the early stages of life [118,119].

Ursodeoxycholic acid (UDCA) can be used to treat IFALD by improving the bile flow [120]. Studies in children reported clinical improvement with full or partial remission of cholestasis, delay of progression of liver fibrosis, shorter duration of cholestasis and lower biochemical liver parameters. However, an important issue is the long-term efficacy of UDCA as withdrawal might be associated with a rebound rise of cholestasis. Aside from this, its use might be limited by diarrhea as this is the most common adverse effect [121,122,123,124].

### 4.4. Small Intestinal Bacterial Overgrowth

Small intestinal bacterial overgrowth (SIBO) is a common side effect in children with PIF on HPN [125]. Though no large consensus exists on how SIBO is defined, it is characterized by an excessive growth of specific microorganisms such as Escherichia coli, Aeromonas, and Klebsiella species in the small intestine [126]. Although duodenal aspirate is considered the gold standard for diagnosing SIBO, lactose or glucose breath test has been suggested for diagnosis, due to its lower invasiveness and better cost effectiveness [79,125]. Anatomical boundaries, particularly the ileocecal valve, are essential for maintaining a balanced microbial community in the human alimentary tract. Multiple studies have confirmed the importance of anatomical changes in the intestine following surgery for PIF causes such as gastroschisis, atresia, midgut volvulus, and necrotizing enterocolitis, and reported high prevalence numbers of SIBO up to 78% [127,128,129]. SIBO is linked not only to surgical complications but also has a higher prevalence in motility disorders such as PIPO [130]. It is hypothesized that dysmotility leads to stasis of intestinal contents, which creates a favorable environment for colonization of bacteria [131]. SIBO symptoms in children vary widely, ranging from mild signs such as abdominal pain, diarrhea, bloating, and flatulence, to severe complications like malnutrition and growth stunting [132]. The primary goals in treating SIBO are addressing the underlying cause, replenishing nutritional deficiencies, and modifying the gut microbiota using antibiotics and possibly probiotics [132]. Antibiotics are most commonly prescribed empirically because duodenal samples are hard to obtain for culture, and it is challenging to identify the specific microorganisms responsible for the infection [132]. Rifaximin is prescribed most often in adults with SIBO as it has activity against both aerobic and anaerobic enteric bacteria presenting minimal adverse effects (e.g., diarrhea) compared to other antibiotics such as metronidazole or amoxicillin-clavulanate [133]. It is proven to be safe to use in children as well [134]. However, an optimal antibiotic regimen including rifaximin in children has not been established yet [133]. In conclusion, no strong recommendations can be made concerning the treatment of SIBO but frequently used medications are metronidazole and amoxicillin-clavulanic acid, see Table 1 [79].

### 4.5. Metabolic Bone Disease

Metabolic bone disease is reported due to decreased bone mineral density, osteoporosis and fractures. Children suffering from congenital enteropathies showed the lowest bone mineral density [135]. This could in part be due to their frequent acid-base and electrolyte abnormalities associated with enteral losses. This may lead to inadequate calcium levels and secondary hyperparathyroidism, which eventually causes decreased bone mineralization [135,136]. A historical risk factor included the excessive infusion of aluminum which plays a role in the development of metabolic bone disease. However, since the introduction of plastic bags for parenteral nutrition instead of glass vials and awareness of high aluminum concentrations in protein hydrolysates, aluminum concentration in parenteral nutrition admixtures has decreased [89,137]. As part of routine follow-up in patients on HPN, regular plasma calcium, phosphorus, active vitamin D and parathyroid hormone, and urinary calcium measurements should be performed. Dual energy X-ray absorptiometry is the primary tool used for measurement of bone mineralization [89]. Treatment with bisphosphonates in a small cohort has been shown to be effective and safe in increasing bone mineral density [138]. More studies should be undertaken to determine the benefit and safety of this therapy. Also, the effect of physical activity on bone mineral density should be investigated in these patients as it is known to improve bone mineral density [139].

### 4.6. Renal Dysfunction

Renal dysfunction and the development of nephrocalcinosis is reported in children suffering from PIF [140,141,142]. The mechanisms through which renal dysfunction develops in patients suffering from PIF are not yet elucidated. However, it is known that children with PIF are prone to dehydration which in time could lead to renal impairment. Serum creatinine is known to be a poor marker for renal function, especially in children with PIF who often have an altered body composition with a decreased fat-free mass index [143]. The estimated glomerular function rate using the combined creatinine and cystatin C Schwartz formula, eGFRcr + cyst, is reported to be the most accurate to assess renal function in children receiving home PN. This measurement combines creatinine GFR, which causes overestimation, and cystatin C GFR, which causes underestimation, making it more accurate for the estimation of renal function [144]. Nephrocalcinosis has been observed in up to 38% of patients suffering from PIF but has not been shown to be associated with a decrease in estimated glomerular filtration rate over a two-year period [140].

### 4.7. Growth

With regards to growth, children suffering from PIF are known to be shorter than their healthy peers with up to 50% of patients growing below target height [135]. Chronic inflammation has been shown to play a role in the shorter stature of patients, but the exact pathophysiology is yet not well understood [135]. Furthermore, children with PİF are shown to have altered body composition with an increased fat mass index and decreased fat free mass index [135,143]. Assessment of body composition in this patient group on a routine basis in addition to standard anthropometrics as part of standard care would allow for a more tailored approach that could help optimize not just height and weight but overall body composition and consequently health.

### 4.8. Health-Related Quality of Life

Children and their parents often report decreased health-related quality of life [11,105,145,146,147,148,149,150] A recently published study on the long-term development of health-related quality of life in patients suffering from PIF showed no change in health-related quality of life over long-term treatment [151]. No association between underlying disease or the number of days patients received parenteral nutrition per week and health-related quality of life was observed [151]. However, this study reported that children aged 5 to 12 years report lower health-related quality of life and greater fatigue than the general population. These findings reflect that active chronic disease patients suffer from their condition and its impact on daily life. Interventions aimed at increasing health-related quality of life, such as regular screening of quality of life through validated questionnaires, should be offered to both parents and patients. Another study about parent–child interaction in pediatric PIF suggested that (self-reported) parent–child interaction in PIF patients and HPN graduates was not significantly different as compared to healthy peers, and unrelated to clinical characteristics. However, aspects of parent–child interaction were correlated to the child’s emotional and behavioral functioning, potentially being a target for prevention or intervention in children with diminished emotional and behavioral functioning [152]. While quantitative studies provide data on the prevalence and severity of psychological distress and quality of life issues, they often lack depth in explaining the underlying reasons and contextual factors contributing to these outcomes. Qualitative research, on the other hand, offers a more nuanced and in-depth exploration of the lived experiences of parents. By integrating findings from both quantitative and qualitative studies, a more comprehensive understanding of the experiences of patients and their families, including parents and siblings, will emerge.

Pediatric chronic intestinal failure is associated with a high risk of feeding difficulties due to enteral tube feeding or prolonged periods of nil per os [153]. Eating, as an important social life skill, may be impaired by eating disorder behavioral characteristics, resulting in a great impact on the quality of life [154]. Therefore, development of healthy eating behaviors, if possible, should be promoted by intestinal failure teams.

## 5. Future Perspectives

Research into the improvement of supportive care for HPN, such as prevention of parenteral nutrition-related complications and improvement of quality of life, remains paramount. Due to the low prevalence of these rare disorders, international collaboration is necessary to investigate the outcomes associated with intestinal failure. A recently launched intestinal failure registry will enable global comparison of different therapies [155]. Furthermore, this potentially large dataset will enable health care professionals to identify patient characteristics associated with successful therapy in order to provide personalized healthcare. European collaboration in the ESPGHAN network of intestinal failure and transplant in Europe (NITE) and the European Reference Network for rare Inherited and Congenital (digestive and gastrointestinal) Anomalies (ERNICA) also provide the opportunity for international collaboration. As patient care will become more and more centered in specialist centers, the future collaboration of these centers will provide valuable insights.

Patient- and/or parent involvement in clinical care and research for chronic conditions play a crucial role as they will represent the true interests and preferences of those affected. Therefore, ERNICA collaborates with patient representatives who are involved in the decision- and opinion-making structures for research and clinical care.

### 5.1. Outcomes in Research

PIF in children is a rare clinical entity, which impedes high quality research in this field. Scarce research is available, and the majority of the performed research is of small sample size. In addition, great heterogeneity in reported outcomes exists, which leads to difficulty in synthesizing and applying the results of different studies [156]. Therefore, a core outcome set for research in pediatric PIF was created. A core outcome set is a standardized set of outcomes ratified by key stakeholders, including patients, parents and health care professionals, which should be measured in all future studies on this topic [157]. The core outcome set for pediatric PIF consists of 10 outcomes important for all key stakeholders: weaning from parenteral nutrition, growth, mortality, central line-related infection, central line longevity, sepsis not related to central line infection, central line-related thrombosis, intestinal failure-associated liver disease, (serious) adverse events, and health-related quality of life. Usage of at least two of these outcomes will minimize outcome heterogeneity and enhance the value of evidence synthesis [42].

### 5.2. GLP-2

Recently, promising medical therapies have become available for children suffering from short bowel syndrome. Glucagon-like peptide (GLP) 2, a naturally occurring hormone which is produced in the terminal ileum and colon by enteroendocrine cells, is known to delay gastric emptying and induce epithelial proliferation through several mediators including epidermal growth factor and insulin-like growth factor 1 [2,158]. Teduglutide, a GLP-2 analogue, was associated with a significant reduction in volume and calories administered via parenteral nutrition (PN) when compared to standard care during a 24-week open-label, controlled trial in a group of 59 children with SBS. Five children were weaned from PN during this study. The number of days patients received PN declined on average by one day for patients receiving teduglutide [159]. Recent Spanish data from routine usage of teduglutide have shown that 12/17 children with SBS with remaining bowel length < 150 cm and with no changes in PN in the last 3 months, who received teduglutide could be weaned from PN after one year [160]. A recent review spanning both aforementioned trials reported that out of 223 children, a total of 36 patients achieved enteral autonomy (16%) after a median of 24 weeks of treatment (IQR: 24–48 weeks) and 149 patients (67%) showed a reduction in PN needs in terms of volume, calories, or hours per day [161]. Overall, teduglutide was associated with mild gastrointestinal side effects such as nausea and abdominal discomfort but was overall well tolerated. Additionally, a recent trial involving adults with short bowel syndrome and intestinal failure (SBS-IF) assessed the impact of teduglutide on quality of life (QoL) among patients dependent on PN. This placebo-controlled study included 86 patients, 43 per group. A significant improvement in SBS-QoL scores was observed in teduglutide-treated subgroups with higher baseline PN volume. Patients with the highest baseline PN volume showed a reduction in SBS-QoL sum score by −27.3 points (95% CI: −50.8 to −3.7). These significant improvements in QoL suggest that teduglutide could potentially also be beneficial for the quality of life in children [162]. Although teduglutide seems promising in children with SBS, it should be emphasized that strict criteria to be eligible to start treatment should be clear. Only patients considered to be incapable of advancing enteral nutrition, after being treated in a multidisciplinary team and after considering all nutritional options, should be treated [159]. In addition to its possibility to improve intestinal adaptation and quality of life, it is also presumed to be eventually more cost effective compared to the standard of care in children with SBS in the future [163]. Currently, evaluation of the cost effectiveness of using teduglutide, with costs estimated to be ∼$400,000 per patient per year, did not show a cost-effective addition to the treatment of pediatric SBS. Cost-reducing strategies, including alternative dosing regimen, should be identified [164].

For example, other GLP-2 analogues are currently under development, with ongoing adult trials potentially expanding treatment options for pediatric SBS in the future. The main other analogues being investigated are apraglutide and glepaglutide in clinical trials, both characterized by their long-acting profile [165,166]. This will offer an advantage as a less-frequent dosing regimen could potentially improve patient compliance and convenience, especially in children in whom daily subcutaneous injections are not preferable.

### 5.3. Transition

Past decades, mortality and morbidity have decreased due to improvements in multidisciplinary, nutritional, surgical and pharmacological care. Children are now able to grow into adulthood which requires transfer from pediatric to adult health care. A recently published transition protocol created by experts from the Intestinal Failure working group of European Reference Network for Rare Inherited Congenital (gastrointestinal and digestive) Anomalies and the Network of Intestinal Failure and Intestinal Transplant in Europe group—ESPGHAN—provides practical guidance for transition which will provide a more structured, optimal transition process [167]. It is advised to use this protocol as a formal checklist that can be placed in the patient’s chart to review and track the transition process by PIF team members.

## 6. Conclusions

PIF is a rare and heterogeneous condition characterized by the inability of the patient’s intestine to adequately absorb the required fluids and/or nutrients for growth and homeostasis. As a result, patients will become dependent on HPN. HPN-associated complications for children with PIF include catheter-related bloodstream infections, catheter-related thrombosis, intestinal failure-associated liver disease, small intestinal bacterial overgrowth, metabolic bone disease and renal impairment. Treatment of PIF has markedly improved with a great reduction in morbidity and mortality. Despite advances in curative and supportive treatment, further research is necessary to improve nutritional, pharmacological and surgical care and prevention of complications associated with HPN use.

## Figures and Tables

**Table 1:** Medical therapies for intestinal failure.

Medication	Type of Intestinal Failure	Reason to Use	Use for Pediatrics/Adults/Both	Evidence and References	Comment and References
1.1. Antisecretory agents					
1.1. Histamine H2 receptors	I, II, III	Reduction in hyperacidity after massive resection.	Both	Level I [61]	
1.2. Proton-pump inhibitors	I, II, III	Reduction in hyperacidity after massive resection.	Both	Level I [61].	- Altering the fecal gut microbiota. - Causing hypomagnesaemia. [62,63].
1.3. Opioid receptor agonist	I, III	Prolonged intestinal transit time.	Both	Level I [64,65]	Increasing duodenal muscle tone and inhibiting propulsive motor activity. Side effects: fatigue, dizziness, nausea and vomiting. Loperamide associated with less side effects compared to codeine.
1.5. Bile acid sequestrants	I, II, III	Bile salt malabsorption after terminal ileal resection.	Both	Level IIa	Reducing absorption of fat-soluble nutrients.
1.6. Somatostatin analogue	I	Reduction in gastric acid and secretion of pancreatic enzymes. Reduction in gastric emptying, gall-bladder contractions and ileal and longitudinal muscle contractions.	Both	Level IIb [66,67]	Glucose levels assessment and blood pressure monitoring whilst on an octreotide infusion is advised.
1.7. Enkephalinase inhibitor	I	Decrease in intestinal secretion and increase in absorption.	Both	Level I [68]	No change in gastrointestinal transit times in rat or mice or healthy human volunteers on racecadotril (enkephalinase inhibitor). [69,70,71]
1.8. α2-receptor agonist	I, II	Stimulation of α2-adrenergic receptors on enteric neurons that also reduce gastric and colonic motility and intestinal fluid secretion.	Adults	Level I [72].	No significant benefit of clonidine (α2-receptor agonist) on volume output reported.
2. Adjunctive absorptive agents					
2.1. Fibers (e.g., psyllium seeds)	I, II, III	Improvement of feces consistency.	Both	Level IIa [73,74]	
2.2. Pancreatic enzymes	I	Increase in intestinal digestion (especially fat) in pancreatic atrophy and exocrine insufficiency.	Both	Level III expert opinion	Proven fat malabsorption (preferably assessed by BOMB calorimetry). [75].
3. Prokinetic agents					
3.1. Serotonin 5-HT4 agonist	II	Delayed gastric emptying.	Both	Level IIb [76,77]	Prucalopride is safe and effective in children. Patients treated with cisapride (serotonin 5-HT4 agonist) require careful cardiac monitoring because of corrected QT prolongation, risk of cardiac dysrhythmias and deaths.
3.2. Erythromycin, clarithromycin, amoxicillin–clavulanic Acid	II	Pro-motility effect.	Both	Level IIa	
3.3. Acetylcholinesterase inhibitor	II	Pro-motility effect.	Both	Level IIb [78].	Pyridostigmine and neostigmine are safe and effective in children in case series.
4. Antibiotic agents					
Mostly used: Rifaximin, Metrodinazole and Amoxicillin/Clavulanic Acid	I, II, III	Treatment of small-intestinal bacterial overgrowth.	Both	Level IIa [79].	- Risk of fungal infections, antimicrobial resistance, or Clostridium difficile infection. - Functional ileocecal valve is of influence for maintaining a balanced microbial flora. - Risk of altering fecal gut microbiota.
5. Probiotic agents					
Many strains and dosages over the counter are available	I, II, III	No evidence of benefit in small studies; risk of bacterial translocation leading to sepsis.	Both	Level IIa [80].	High risk of adverse effects in immunocompromised and debilitated patients.
6. Growth factors					
GLP-2 analogues	I	Stimulation of intestinal adaptation and absorption, decreasing fecal losses.	Both	Level I [81].	Teduglutide is licensed for use in adults and children with short bowel syndrome.

Pediatric Chronic Intestinal Failure. Canadian Task Force on the Periodic Health Examination’s Levels of Evidence. Level: Type of evidence. I: 1 or more RCT. IIa: Well-designed cohort or case-control study. IIb: Time series comparisons or dramatic results from uncontrolled studies. III: Expert opinions. Type intestinal failure: I. Short bowel syndrome. II. Motility disorder. III. Congenital enteropathy.
